# Unveiling the Chilaiditi Syndrome: A Case Report and Management Implications

**DOI:** 10.7759/cureus.57483

**Published:** 2024-04-02

**Authors:** Ibrahim Kamel, Yusuf Yalcin, Reid Ponder, Ibrahim Elkhawas, Zeeshan Solangi

**Affiliations:** 1 Internal Medicine, Steward Carney Hospital, Boston, USA; 2 Radiology, Tufts University School of Medicine, Boston, USA; 3 Pulmonary Critical Care, Baystate Medical Center, Springfield, USA

**Keywords:** lactic acidosis, computed tomography, colonic interposition, radiologic finding, chilaiditi syndrome

## Abstract

The Chilaiditi syndrome is when the radiologic Chilaiditi sign, defined by the interpositioning of the colon between the liver and diaphragm, becomes complicated by clinical symptoms such as respiratory insufficiency or bowel obstruction. We present the case of a 70-year-old male with a history of depression, anxiety, gastroesophageal reflux disease (GERD), and post-polio syndrome, who presented with left shoulder pain, chronic weakness, and dizziness. Initial evaluation revealed hypotension and elevated lactic acid, attributed to dehydration. Further imaging identified a Chilaiditi sign, thus raising suspicion of small bowel obstruction and the Chilaiditi syndrome. Despite conservative management, the patient continued to experience elevated lactic acid levels, prompting a computed tomography (CT) angiogram to rule out bowel ischemia. No acute intra-abdominal pathology was identified, and the patient improved with hydration and bowel rest. This case highlights the challenges in diagnosing and managing the Chilaiditi syndrome in the setting of chronic comorbidities.

## Introduction

The Chilaiditi sign is a rare, incidental radiologic finding secondary to colonic interposition between the liver and diaphragm. The Chilaiditi syndrome then arises if a patient develops associated clinical symptoms. Current literature reports a sign incidence between 0.025% and 0.28% on plain film, although computed tomography (CT) evaluation is more sensitive with incidence estimates between 1.2% and 2.4% [1-3]. The etiology of the Chilaiditi sign involves both congenital and acquired factors, ranging from ligament abnormalities, right diaphragm paralysis, chronic constipation, redundant colon, liver cirrhosis, and multiple pregnancies [4]. The mechanism is poorly understood, although it is thought to depend on both a hypermobile intestine and an enlarged subdiaphragmatic space [5].

As the Chilaiditi sign and syndrome are characterized by apparent subdiaphragmatic air, several dangerous conditions can present similarly on imaging. This list includes pneumoperitoneum, subphrenic abscess, diaphragmatic hernia, and, in the context of trauma, diaphragmatic rupture [6-8]. Management for these etiologies is surgical and frequently urgent. In contrast, the Chilaiditi syndrome can be a self-resolving or chronic entity and could therefore be managed with a more conservative approach [9]. We present a case of a 70-year-old male who presented with left shoulder pain, chronic weakness, and dizziness and was found to have hypotension, elevated lactic acid, and a Chilaiditi sign. We discuss our clinical reasoning and management of this patient.

## Case presentation

**Figure 1:**
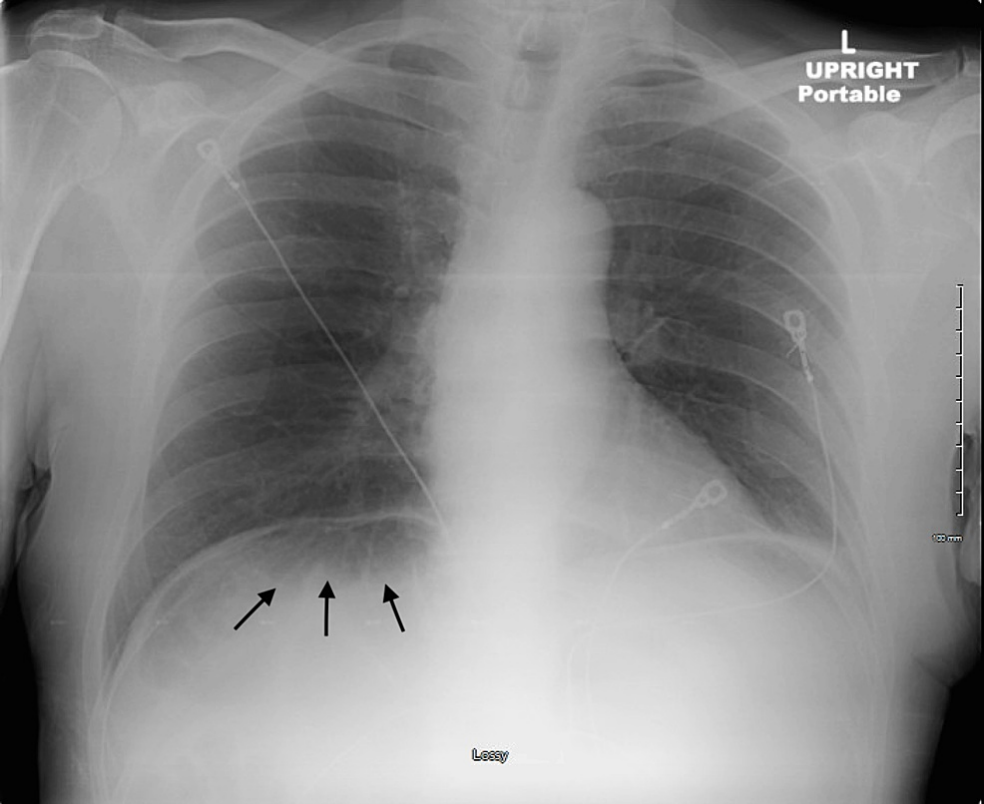
Chest X-ray Chest X-ray showing bowel loops under the diagram. The black arrows pointing at the bowel loops

A 70-year-old male with a history of depression, anxiety, gastroesophageal reflux disease (GERD), and post-polio syndrome presented to our emergency department with left shoulder pain, chronic weakness, and dizziness. He was found to be hypotensive by a visiting nurse and brought to the emergency department. Initial evaluation at our center revealed hypotension (87/61 mmHg), elevated lactic acid (4.6 mmol/L), and normal oxygen saturation. The chest X-ray shown in Figure 1 was unremarkable for acute cardiopulmonary pathology but was remarkable for subdiaphragmatic air.

**Figure 2:**
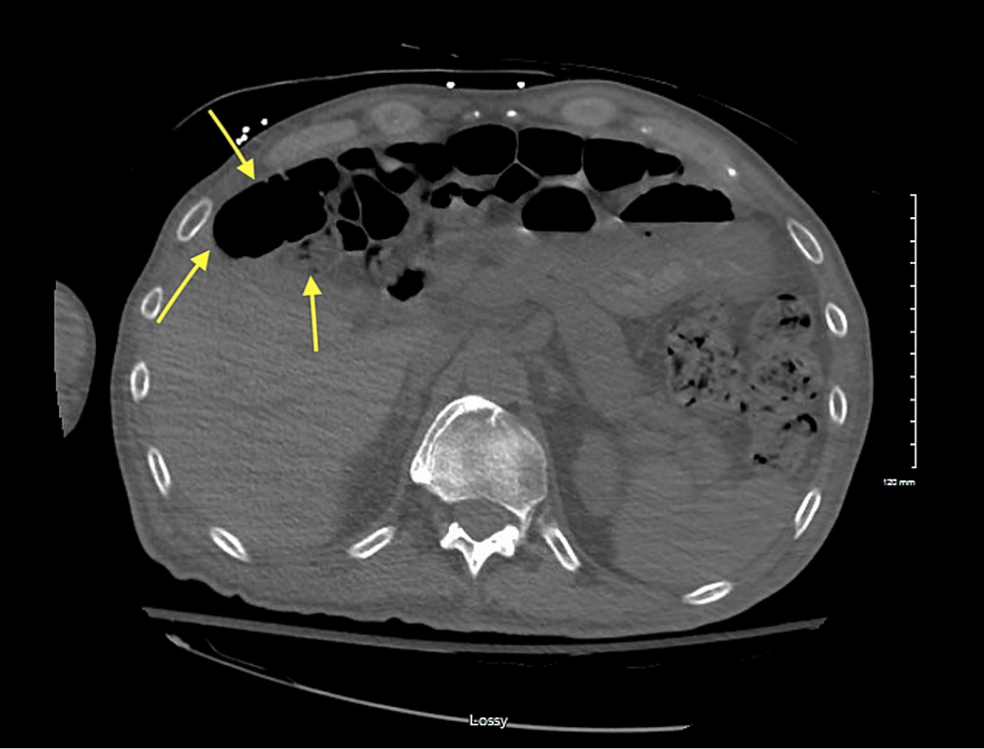
Abdominal CT one year prior to presentation Image showing CT scan of the abdomen with the bowel loops between the abdominal wall and the liver. The arrows pointing at the bowel loops CT: computed tomography

Further review demonstrated overlying bowel loops, suggesting that this air was intraluminal and secondary to an interposed intestine. The patient’s chart was reviewed for the anatomical variant of the Chilaiditi sign, and a CT of the abdomen from one year prior showed haustral markings between the abdominal wall and liver, consistent with the Chilaiditi sign. The results of this study are shown in Figure 2.

**Figure 3:**
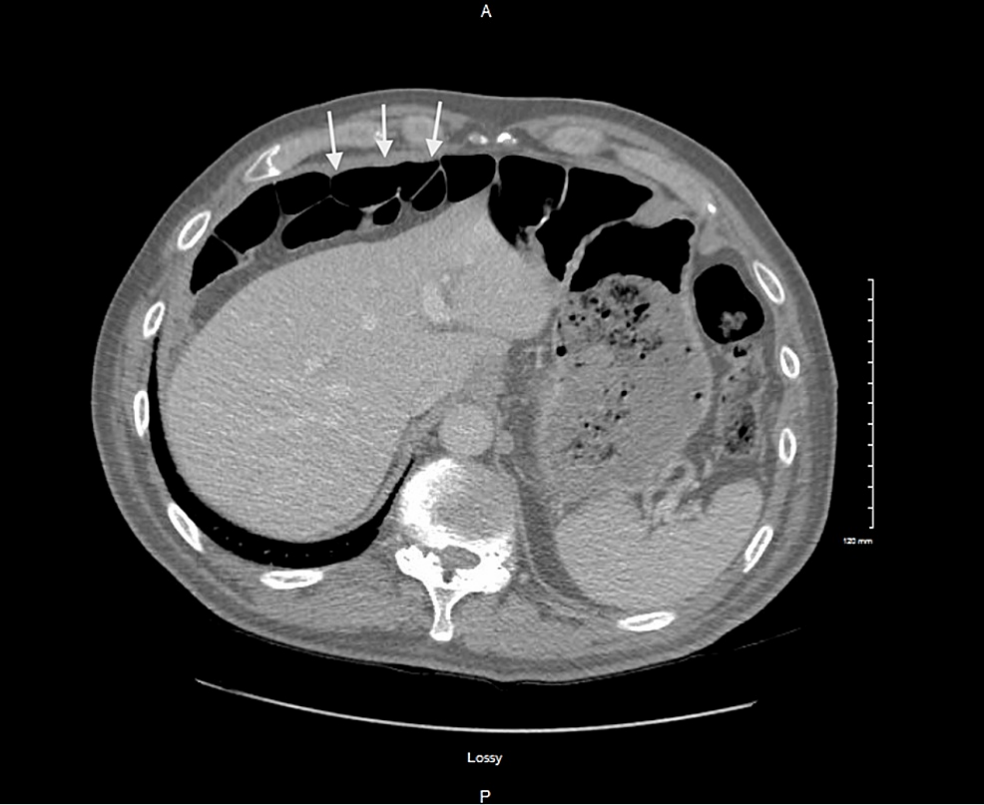
Abdomen/pelvis CT of the abdomen/pelvis with white arrows pointing at the bowel loops interposed between the abdominal wall and the liver, which are dramatically changed from the previous study CT: computed tomography

Further evaluation showed no clinical signs of infection, abdomen that was soft, and no tenderness or distention. The patient improved with IV hydration. However, lactic acid levels continued to increase, prompting a CT of the abdomen and pelvis shown in Figure 3, which again revealed the Chilaiditi sign with suspicion of small bowel obstruction.

Notably, this study contained more interposed bowel loops compared to the CT from one year prior. Surgery recommended bowel rest and close monitoring, especially given the patient’s deconditioning and overall frailty.

**Figure 4:**
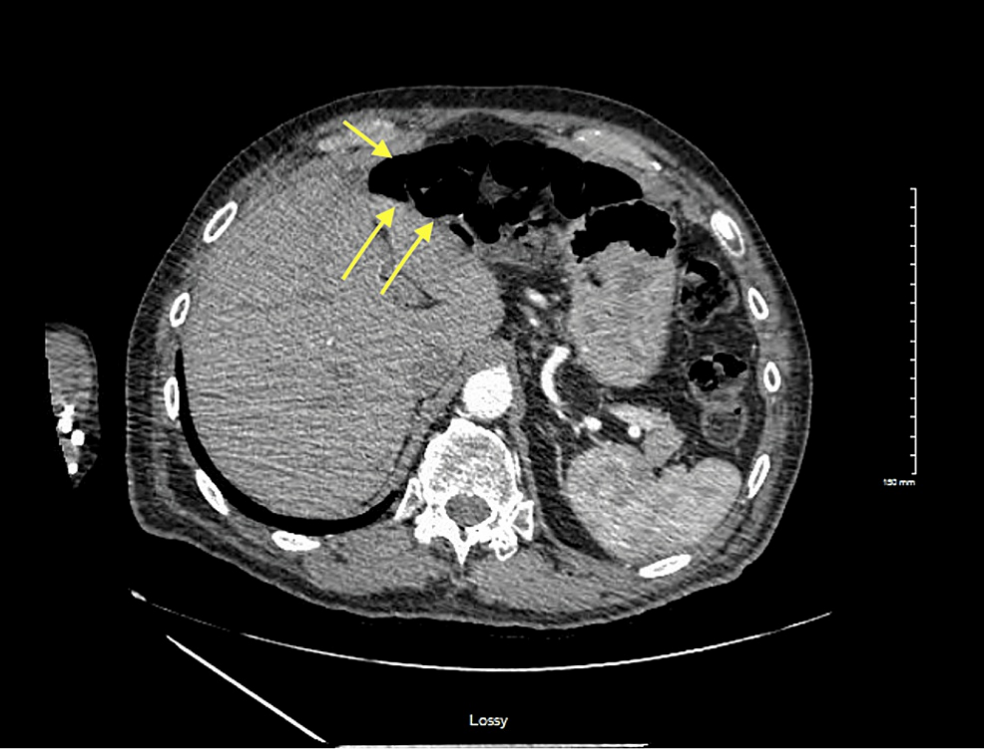
CT angiogram of the abdomen/pelvis Abdominal CT angiogram reveals the bowel loops receding and not interposed between the liver and abdominal wall CT: computed tomography

Despite bowel rest and hydration, lactic acid levels fluctuated, necessitating a CT angiogram to rule out bowel ischemia. The results of this test are shown in Figure 4.

No acute intra-abdominal pathology was identified, and the patient improved with conservative management.

## Discussion

On admission, our patient was found to be hypotensive and have lactic acidosis, concerning for an ischemic or infectious process and potential sepsis. Imaging was obtained and demonstrated subdiaphragmatic air. As previously mentioned, the etiologies of subdiaphragmatic lucency are broad and potentially emergent. This case underscores the importance of integrating radiologic findings with clinical status: employing surgical management could have increased morbidity for our patient, given his baseline frailty. The chart review demonstrated that the apparent air was a chronic process and not due to an acute pathology such as bowel ischemia and perforation, even with the elevated lactate. The patient was noted to have the anatomic variant of the Chilaiditi sign, a critical distinction that led to conservative treatment.

The prevalence of the Chilaiditi sign is highest in the elderly [10]. Considering the aforementioned drivers of the Chilaiditi sign, intestinal hypermobility and an enlarged subdiaphragmatic space, the reasons for this may be generalized frailty and colonic redundancy secondary to comorbid constipation, a condition that correlates positively with age [10,11]. We considered possible etiologies for our patient’s Chilaiditi sign, and given his baseline muscle weakness, we proposed that incomplete diaphragmatic excursion could have led to subdiaphragmatic space enlargement. Meanwhile, chronic constipation may have contributed to colonic redundancy and displacement.

While our case had the benefit of prior images, an isolated finding of subdiaphragmatic air is often concerning. Therefore, it is important to consider imaging techniques that may increase suspicion of the Chilaiditi sign, as many emergency department patients will present without baseline examinations. Typical features include haustral markings that surround the subdiaphragmatic air and a lack of positional change of the subdiaphragmatic air when the patient moves [12]. This second characteristic is because free, extraluminal air is gravity-dependent, whereas the Chilaiditi syndrome air is contained by the interpositioned colon. Even if the patient is in the left lateral decubitus position, the Chilaiditi sign air will still be trapped in the subdiaphragmatic space. Such imaging pivot points would be of paramount importance in the presented case, as the patient’s age, chronic weakness, frail state, and lactic acidosis would have made him a poor surgical candidate. Moreover, the high surgical risk is an issue likely common to many Chilaiditi syndrome patients, considering its elderly predilection [10].

The Chilaiditi syndrome has a diverse presentation, including respiratory distress and angina-like episodes [6]. The appropriate treatment therefore considers symptom severity, with critical manifestations such as those complicated by bowel obstruction prompting surgical intervention [13]. The chronicity and frequency of associated symptoms also have a role in dictating management. A recent case report proposed staging and treatment protocols for the Chilaiditi syndrome, with recommendations dependent on these factors [14]. Following their proposed algorithm for our case would have led to the same course of conservative treatment; however, such tools are useful in supporting management decisions and for settings in which the syndrome recurs. After inpatient stabilization, mild instances of the syndrome could also be aided by physical activity after discharge, as exercise could promote intestinal peristalsis and symptom resolution [15]. Meanwhile, for severe cases, additional workup could include Gastrografin enemas, CT with endoluminal contrast, and colonoscopy [14]. Reported successful surgical interventions for these instances include total or partial colonic resection combined with hepatopexy [6,13]. Some patients have even required diaphragmatic intervention [16,17].

## Conclusions

In summary, the Chilaiditi sign, a rare radiologic finding caused by colonic interposition between the liver and diaphragm, can progress to the Chilaiditi syndrome when clinical symptoms arise. Despite its low incidence, computed tomography is more sensitive in detection. Its etiology involves congenital and acquired factors, often affecting elderly individuals. Differential diagnosis is crucial due to imaging manifestations resembling serious conditions. Our case highlights the importance of accurate diagnosis, as conservative management effectively resolved symptoms, avoiding surgery in a high-risk patient. Tailored treatment strategies, guided by symptom severity and recurrence, are essential, with recent literature proposing staging and treatment protocols. Understanding the diverse presentations and management options is critical for optimizing outcomes, especially in elderly patients with multiple comorbidities.
